# P-1569. Microbiome Derived Stool Metabolites Predict E. faecium Expansion in Hospitalized Patients

**DOI:** 10.1093/ofid/ofaf695.1749

**Published:** 2026-01-11

**Authors:** Wesley J Hudson, Ramanujam Ramaswamy, Dinanath Sulakhe, Mark D’Souza, Ashley Sidebottom, Victoria Burgo, Matthew Odenwald, Sabrina Imam, Bhakti Patel, Ann Nguyen, Eric Pamer, Christopher Lehmann

**Affiliations:** The University of Chicago Pritzker School of Medicine, Chicago, IL; University of Chicago, Chicago, Illinois; University of Chicago, Chicago, Illinois; University of Chicago, Chicago, Illinois; University of Chicago, Chicago, Illinois; University of Chicago, Chicago, Illinois; University of Chicago, Chicago, Illinois; University of Chicago, Chicago, Illinois; UChicago Medicine, Chicago, Illinois; University of Chicago, Chicago, Illinois; University of Chicago, Chicago, Illinois; University of Chicago, Chicago, Illinois

## Abstract

**Background:**

Vancomycin resistant *Enterococcus faecium* (VRE) poses a distinct threat to hospitalized patients.(CDC, 2022) Expansion of VRE within the gut microbiome is closely linked with invasive infections in multiple hosts.(Lehmann et al., 2024; Taur et al., 2012) Stool microbial metabolite measurement offers a new diagnostic avenue to rapidly identify VRE colonization. (Lehmann et al., 2024) We developed a machine learning model using stool metabolite measurements to predict *E. faecium* expansion in patients.

**Table 1.**
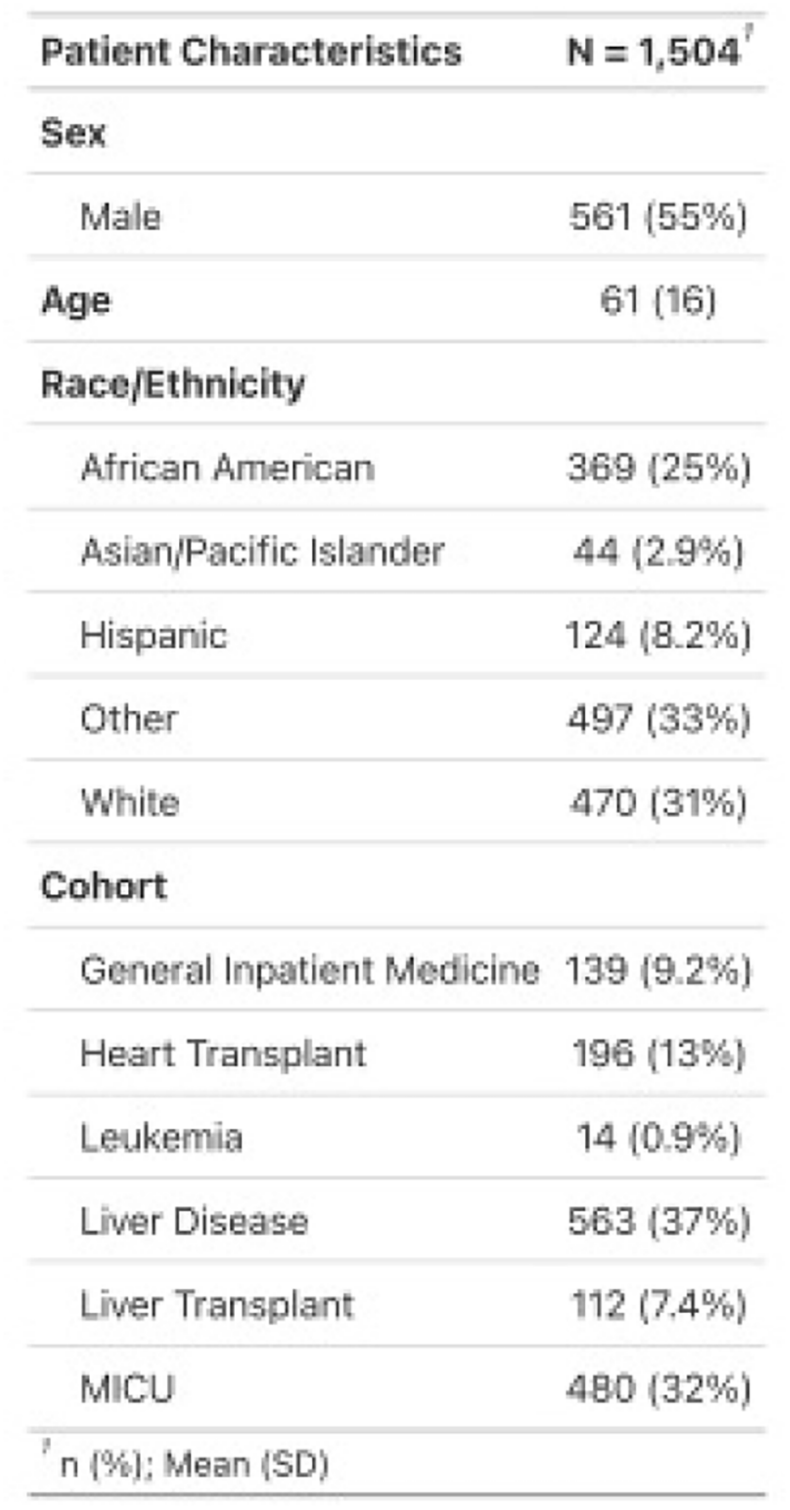
Patient Characteristics. Demographic information of the patients whose samples were utilized to train and test this model.

**Figure 1.**
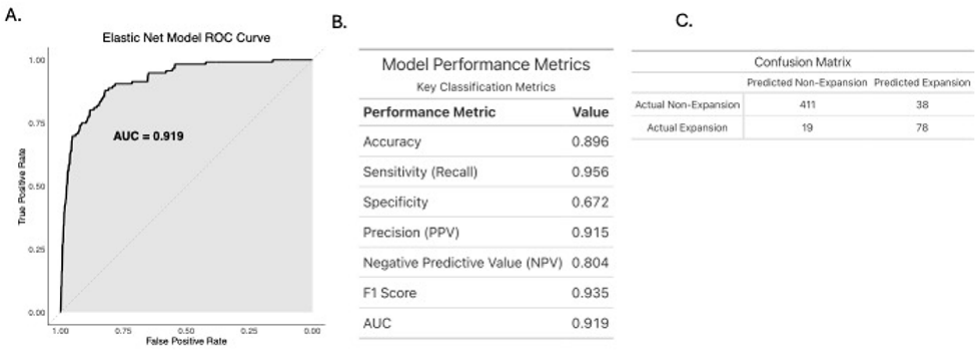
Overall performance of the Elastic Net Model. A. Receiver operator characteristic plot showing true positive vs false positive rates. This demonstrates an area under the curve of 0.919. B. Table displaying a variety of model performance metrics. Notably, this model demonstrates a high F1 score of 0.935 as well as positive and negative predictive values of 0.915 and 0.804 respectively. C. Confusion Matrix of model performance on unseen test data.

**Methods:**

Using qualitative stool metabolite concentrations as measured by targeted GC and LC-MS analysis paired with microbiota composition obtained via shotgun metagenomic sequencing, we generated, tuned, and evaluated an elastic net-based machine learning model to predict stool expansion of *E. faecium* over 30% relative abundance.(Kuhn, 2008) Collinearity and multicollinearity analysis of the metabolites was performed using Spearman’s Rank-Order method, and Variance Inflation Factors respectively. Following hyperparametric tuning of this model, test data was used to evaluate the model’s performance. The selected classification cutoff for the model maximized the F1 score based on precision and recall.

**Figure 2.**
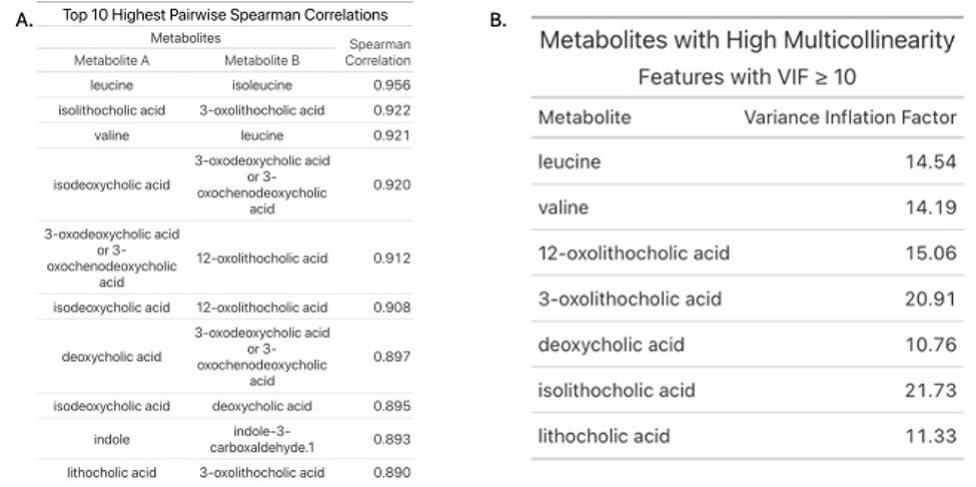
Collinearity Assessment of Stool Metabolites. A. Top 10 pairwise Spearman Correlations are listed with both respective metabolites and the absolute value of their correlation. B. All metabolites that demonstrate high multicollinearity (VIF>10) are shown with their respective variance inflation factor.

**Results:**

2738 stool samples from 1504 patients were included from 6 observational clinical studies on liver disease, liver transplant, heart transplant, critical care, internal medicine, and leukemia patients.(Dela Cruz et al., 2023; Lehmann et al., 2024; Odenwald et al., 2023; Stutz et al., 2022) (Table 1) The model predicted *E. faecium* expansion with an accuracy was 0.894, sensitivity of 0.949, precision of 0.919 and AUC of 0.917. (Figure 1.)

**Conclusion:**

This work provides a proof of concept that stool metabolites can identify pathogen expansion in the gut. The approach could aid in early diagnosis leading to better outcomes. It could also identify key inhibitory metabolites as future therapeutic candidates. Future work will identify the metabolites with highest predictive value and apply this method to other gut pathogens such as *Klebsiella pneumonia*.

**Disclosures:**

Bhakti Patel, MD, CHEST: Board review course director|Merck: Wrote medical chapters

